# Degradation Behavior of Polymers Used as Coating Materials for Drug Delivery—A Basic Review

**DOI:** 10.3390/polym13081272

**Published:** 2021-04-14

**Authors:** Anita Ioana Visan, Gianina Popescu-Pelin, Gabriel Socol

**Affiliations:** Lasers Department, National Institute for Lasers, Plasma and Radiation Physics, 077190 Magurele, Ilfov, Romania; gianina.popescu@inflpr.ro

**Keywords:** degradation, biopolymers, thin films, in vitro characterization techniques, bioreactor

## Abstract

The purpose of the work was to emphasize the main differences and similarities in the degradation mechanisms in the case of polymeric coatings compared with the bulk ones. Combined with the current background, this work reviews the properties of commonly utilized degradable polymers in drug delivery, the factors affecting degradation mechanism, testing methods while offering a retrospective on the evolution of the controlled release of biodegradable polymeric coatings. A literature survey on stability and degradation of different polymeric coatings, which were thoroughly evaluated by different techniques, e.g., polymer mass loss measurements, surface, structural and chemical analysis, was completed. Moreover, we analyzed some shortcomings of the degradation behavior of biopolymers in form of coatings and briefly proposed some solving directions to the main existing problems (e.g., improving measuring techniques resolution, elucidation of complete mathematical analysis of the different degradation mechanisms). Deep studies are still necessary on the dynamic changes which occur to biodegradable polymeric coatings which can help to envisage the future performance of synthesized films designed to be used as medical devices with application in drug delivery.

## 1. Introduction

**Figure 1:**
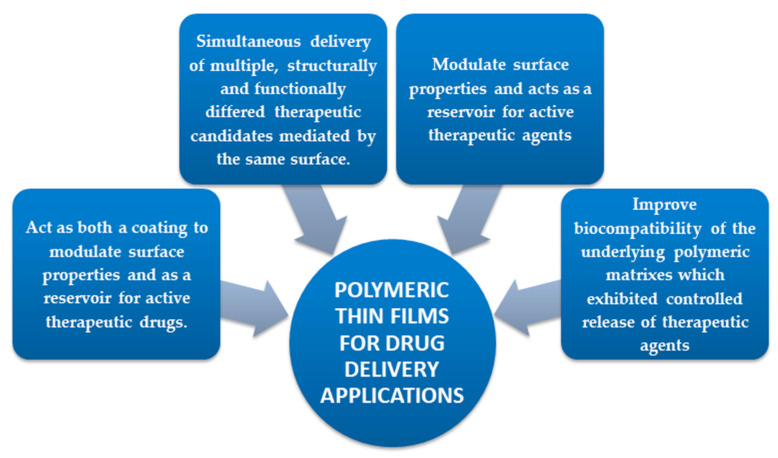
Benefits of surface biofunctionalization with polymeric coatings.

Undoubtedly, biodegradable polymers have a major role in the functionalization of biomaterials and medical devices (MD), due to their capability to be degraded and eliminated in time under physiological conditions [1]. It is well known that biomaterials interact with biological systems through their surfaces [2,3] and so that it is of great importance to control or tune the surface properties of MD, helping them to safely and easily integrate into the host tissues [4]. In this context, for the drug delivery specific applications, several advantages are obtained by surface functionalization of MD with polymeric coatings (P.C.). Based on their degradable features, they can incorporate drugs which are delivered by predetermined release profiles at a desired site of action while being easily eliminated by the body or even replaced in time by tissues [1]. The potential of surface functionalization with P.C. (Figure 1) could solve the critical problems of used polymer amount supply associated with processing time. Moreover, thin film deposition techniques are designed to ensure the MD functionalization by improving the surface features with a newly formed polymeric platform. Concretely, polymeric thin films present an amazing versatility in the chemical groups which can help control biomaterial-tissue interactions and also possess the required mechanical properties due to substrate [5]. Plenty of deposition techniques, such as Langmuir-Blodgett deposition [6], spin coating [7], sputtering [8], chemical vapor deposition [9,10], electrochemical deposition [11], spray coating [12], or advanced laser techniques (e.g., matrix assisted pulsed laser evaporation [13]), chemical grafting [14], self-assembled monolayers [15], surface-tethered polymers (polymer brushes) [16], dip-coating [17,18], electrophoretic deposition method [19], or multilayer [20,21], were extensively employed to fabricate P.C.

Each deposition technique may show particular advantages in a given situation and so that the choice of the proper coating fabrication route should be therefore determined based on the desired medical application, the workability of polymer, and its physico-chemical properties [22].

An excellent option for the improvement of the metallic implants is the tailor-made coating (for accelerated tissue regeneration, with antibacterial properties and/or controlled release), making them more affordable and reducing or even eliminating the need for further surgical revisions [23].

Thin films also present advantages over bulk polymers due to their large surface-to-volume ratios, being suitable for applications requiring enhanced surface interactions. Another benefit of coatings over raw materials is the achievement of application-specific properties that are unattainable in the case of uncoated material or in the raw starting material used to be applied for surface functionalization.

A successfully integrated P.C. functionalized MD supposes a careful corroboration and analysis of polymeric thin films surface properties (such as morphology, micro-and/or nano-scale topography, chemical structure, and composition), with the dynamic phenomena that occur at interfaces (e.g., adsorption, modification, or wetting) [24].

Most of these properties need to be optimized for the achievement of a specific application. The control of the film properties requires well established deposition parameters for each polymeric system and a thorough understanding of the underlying mechanisms of deposition (e.g., nature of deposited polymers, the interactions among process and material parameters) [25]. At the same time, the next step toward more effectively designed devices based on biodegradable P.C. implies a better understanding of their behavior in this form.

For some applications, the development of functionalized P.C. is in close connection with the degradation process. Although in its infancy, the in vitro study of the dynamic changes which occurs in a biodegradable polymeric thin film can be effective in predicting the body’s physiological regulation mechanisms and future performance of designed implantable M.D.

The degradation process of a P.C. employed in drug delivery applications involves the degradation of the whole structure which may contain, besides copolymers, blends of polymers or composites, the active substance, namely the drug. Another aspect to keep in mind is that the individual components that form the coating matrix may have different degradation routes (e.g., one of the compounds may solubilize while the other degrades). Thus, the degradation rate and the drug release can be accordingly tuned.

This review is dedicated to the degradation mechanisms which occur in polymeric thin films with particular highlights on the correlation between raw polymer and the deposition techniques that allow the controlling and tuning of polymer properties and thus the MD functionality.

This review is dedicated to the degradation mechanisms which occur generally in P.C., with particular highlights on the correlation between the properties of the raw polymer and the deposition techniques that allow the controlling and tuning of polymer properties and thus the M.D. functionality.

**Figure 2:**
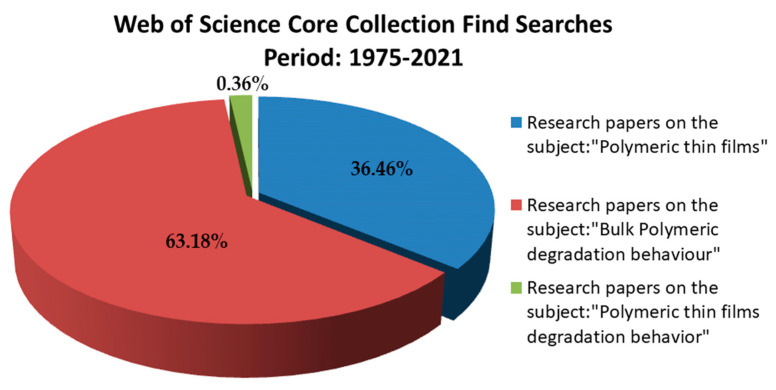
Current state of relevant publications available in the reviewed research field for studies related to the degradation of polymers in form of coatings.

A digital survey based on the criteria described in Figure 2 was performed for the period 2000–2021, using Web of Science (http://apps.webofknowledge.com accessed on 31 January 2021). There is still poor and fragmented understanding of the polymeric thin films degradation behavior as according to Web of Science Core Collection (the research papers were limited to only 99 results). Comparatively, in the same period, 10,031 manuscripts on the polymeric thin films subject emphasize the advantages of applying polymer-based thin layers for MD functionalization. Search terms were put in double-quotes to restrict the search result to the specific phrases. The search field was specified to seek only abstracts, title, and keywords, and was restricted to only ISI journal articles. This statistic fully justifies that the need to investigate (to which are attributed over 17,385 manuscripts) was not sufficiently evaluated.

The difficulty with respect to the degradation behavior of polymers in form of coatings can be also related to the amount of the active substance (e.g., drugs, natural antimicrobial agents, etc.) that can be incorporated into the polymeric matrix correlated with the statistical process of synthesizing reproducible thin films. Other issues encountered in the degradation studies on P.C. are related to the large variety of their physico-chemical features that depends on the deposition methods and the chosen parameters. Moreover, the interdependence between the nature of substrates, the adherence features, and the range of thicknesses leads to a variety of possible combinations to be considered. In order to predict the functionalization and performance of M.D. based on P.C. there is still a place for studying the proposed subject. Research efforts should be further directed toward improving and controlling polymers physico-chemical properties to obtain sustainable coatings for drug delivery applications. As a perspective, personalized composite coating can be tuned according to the patient’s needs, offering the opportunity for operability in the polymer composition and properties.

In this review a brief introduction and classification of degradable polymers used as coatings is given, together with the degradation mechanisms and the factors affecting the process, as well as the fabrication techniques and corresponding testing methods (for both bulk and coatings) and their applications in drug delivery. Furthermore, current research approaches and future perspectives in the application of controlled degradation processes as alternative and viable routes toward enhanced polymer-based coatings’ degradation and functionalization are presented.

## 2. Controlled Drug Delivery Application

The fabrication of MD with functionalized polymeric surfaces which exhibit a controllable rate of degradability in time could be influenced by several factors:

− Both simple polymers or polymeric blends can be used for tuning the degradability rate of the polymeric matrix (the drug can be released immediately or gradually over time depending on the desired application).
− The degradation process can be also controlled by blending or copolymerization.
− Depending on the drugs to be released; polymeric systems can be found in a wide range of medical applications, e.g., orthopedics or drug release (cardiovascular stents, wound healing, skin grafts, absorbable surgical implants, and bone plates).

Compared to the classic release of drugs, the polymeric systems coatings offer the advantage that by optimizing these parameters one can obtain a controlled drug release both as location and as time.

**Figure 3:**
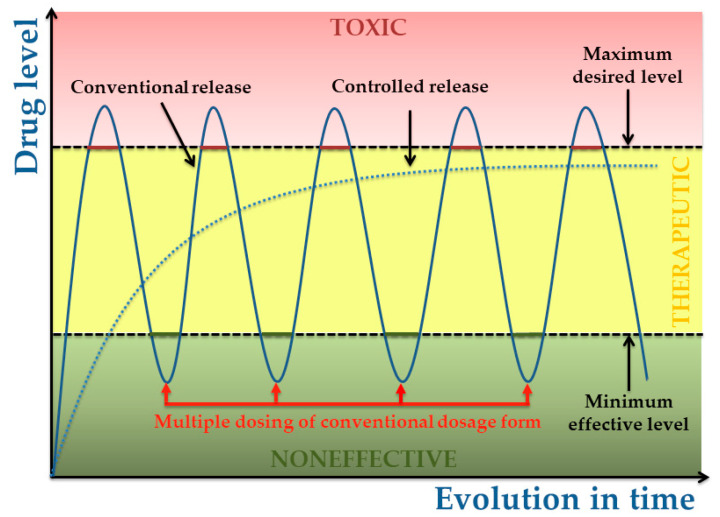
Plasma drug concentration profiles for conventional and controlled release formulations.

The purpose of any drug delivery polymeric system (either bulk or coating) is to provide and maintain a proper therapeutic concentrations of drug at the target biological site during time (Figure 3) [26].

The drug safety and efficacy could be improved by designed personalized drug polymeric system, dose titration, and therapeutic drug monitoring [27]. The release of a drug (controlled rate, slow delivery, targeted delivery) from a surface which is in direct contact with the organism needs to be investigated from a toxicological point of view [27]. Polymeric coatings, known for their unique surface properties as compared to bulk materials, are usually designed to improve the solubility and to assure a chemical stability of drugs, to increase pharmacological activity, and to reduce their side effects [27].

The basic steps in the release of drugs from degradable polymeric systems are:

1. An initial burst due to the dissolution/erosion or diffusion of the drug (the drug release occursby transferring the dissolved medication ingredients through water-filled pores); this kind of release can be of two types: encapsulation or a matrix system.
2. A lag phase.
3. The controlled release of the drug governed by polymer degradation [27].

In the case of the encapsulated dissolution, the release rate is dependent on the thickness and the solubility of the polymeric coating in which was embedded the drug. The mixture of drugs with early or delayed release can be incorporated in the same coating, tablet, or capsule [26].

In the case of a continuous drug release from a polymeric matrix, the diffusion- and degradation-controlled phases must overlap. Therefore, so much more polymer degradation profiles proved important for a controlled release formulation, the kinetic of the drug release being able to be tailored precisely with the help of biodegradable polymers [27].

## 3. Biodegradable Polymers Used as Coatings

### 3.1. Terminologies Background

Taking into account that biodegradation process and degradable polymers are defined in a variety of ways in the literature, it is fully justified the need of a short section regarding the used terminology. In this review, we have adopted the definitions as listed in the IUPAC Compendium of Chemical Terminology [28].

The biodegradable term refers to a biologically assisted degradation process. Concretely, according to IUPAC Compendium of Chemical Terminology, a biodegradable polymer is a polymer susceptible to degradation by biological activity, accompanied by a lowering of its molar mass [28].

The drug release from a polymeric system can be controlled by many mechanisms (e.g., erosion, partitioning, dissolution, swelling, osmosis, targeting, and diffusion) [28]. These mechanisms may act simultaneously or independent at different stages of a delivery process [28,29,30]. It is common for a system or MD to present more than one of them, but the degradation behavior of P.C. is governed by the properties of the main polymer constituents [28,29].

The biodegradation term is explained as a breakdown of a substance catalyzed by enzymes in vitro or in vivo [28,29].

The term bioreactor is associated to an apparatus used to carry out any kind of bioprocess (examples include fermenter or enzyme reactor). A broader definition of the term should include the reactor, i.e., where degradation and solubilization are similar reproduced in simulated environments, such as simulated body fluid (SBF) or phosphate buffered saline (PBS), using the same blood flux rate, etc. [29,30].

In order to be used in medical applications, a biodegradable polymer must fulfill certain criteria [30] such as:

− Non-toxic response after implantation in the body.
− Reasonable shelf life.
− Non-toxic degradation products able to get metabolized and easily eliminated from the body.
− The degradation time should match the therapy process time (e.g., healing, regeneration, or treatment).
− Appropriate mechanical properties for the desired application and the inherence variation in mechanical properties that occurs with the degradation, compatible with the healing or regeneration process.
− Appropriate processability in order to tailor the mechanical properties of MD in correlation with the intended application.

The polymer based coatings designed for controlled release have the advantage of being able to maximize the therapeutic benefit as they do not require replacement or further manipulation and can degrade into non-toxic and soluble components [30].

One should mentioned that the coating term is a general one, referring to a variety of applications from functional ones to healing, meanwhile thin films are used for covering, being obtained especially by deposition techniques [31,32,33].

Moreover, the term thin films refer to layers of material ranging from nanometers to several micrometers in thickness. There is another difference to be highlighted between a thick and a thin film. Concretely, a thick film will typically have a thickness in the range 10–25 μm, surface layers thicker than 1 micron being classified as coating [31,32,33]. However, the real difference between thin and thick films, more than their relative thickness, is the way in which they are synthetized [33]. Thin films are often deposited using vacuum techniques such as sputtering and molecular beam epitaxy, meanwhile thick films are deposited from a solution or paste, which must be dried and then often sintered to produce the final coating [32].

These clarifications are necessary because the thickness may critically influence the interaction of coatings with physiological and body fluids and further targeted effect. Polymers present different degradation rates within the organism and therefore their selection can be tailored to achieve the desired release rates or bioresorption behavior.

Whatever the application or the desired degradation kinetics, it is essential to understand the degradation mechanisms in order to be able to define or guarantee either the stability and/or the controlled degradation rate.

### 3.2. Classification of Biodegradable Polymers

Degradable polymers can be generally classified according to their origin as natural or synthetic polymers. Most of the natural-based polymers are completely biodegradable while the synthetic ones and their blends do not degrade completely [5]. Both are subdivided into different classes based on the main linkages present in their structure.

Depending on their chemical composition, polymers degradation can take place passively by hydrolysis or actively by enzymatic reaction.

**Figure 4:**
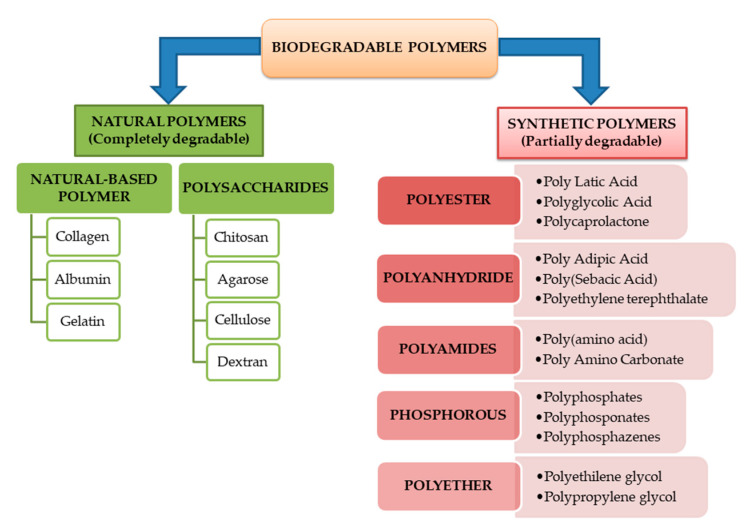
Schematic representation of biodegradable polymers classification after their nature.

In this context, the polymers can be classified taking into account the degradation mode in completely or partially degradable polymers. One could mention here the class of polymers with limited biodegradation capacity. From this category, the ones commonly used as polymer coatings are cellulose and cellulose derivatives (e.g., microcrystalline cellulose). Figure 4 summarizes the general classification of degradable polymers as presented above.

In this subsection, a selection of biodegradable polymers (natural and synthetic ones, including polymers with low biodegradation capability) which can be used as matrices for drug delivery are discussed.

In Table 1 some of the most used polymers in the form of bulk and coatings are described, mentioning for each case the degradation time.

As in the case of P.C. obtaining methods, the degradation capability of bulk polymers can be also influenced by the polymer processing methods (e.g., mechanical mixing, blending, dissolution in co-solvent, use of monomers for polymerization, fine powder mixing) [89]. Polymer physics and chemistry, together with the engineering methods (e.g., mixing, rheology, solid mechanics, reticulation), are important factors in predicting the complex relationships between process method, micro- or macrostructure developed, and the implicit effects of these factors on the final properties of the synthesized polymer [90]. Generally, bulk polymer processing can lead to enhancing the raw materials properties, such as: brittleness, dimensional stability, modulus, chemical resistivity, biodegradability, thermal stability, etc. [90]. For example, the melt fracture of polymers can be reduced by processing (mechanical mixing/blending) which can, in the same time, help to reinforce the particulate [89]. Additionally, in the case of P.C., both composition (including molecular size, chemical branching, cross-linking) and deposition technique (influenced by substrate nature, adherence issues, statistical reproducibility of the process) are critical to the estimated properties of the final functionalized film [90]. The degradation rate of P.C. can be modulated by tailoring the molecular weight, composition, end groups, pore, geometry, and coating thickness, so that the degradation behavior not to vary from one patient to another [90].

In the following, we will briefly approach the specifics of P.C. compared to raw polymers by mentioning some representative literature survey on the characteristic degradation mechanism of the illustrative selected polymer from each category (natural and synthetic, including polymers with low biodegradation capability).

#### 3.2.1. Natural Polymers

Degradation mechanism of one representative polypeptide, PHVB, involves several parameters related to intrinsic physical and chemical properties as emphasized by enzymatic degradation studies [91]. PHBV degradation mechanism is involving the ester group hydrolysis series of reactions initiated by free radicals, cross-linking reactions, and Norrish I and II mechanisms. Copolymerization is one of the methods for improving biodegradability and fine-tuning functional properties. It was shown that increasing hydroxyvalerate content in PHBV can decrease its elastic modulus, melting temperature and tensile strength with higher elongation [92]. In the same way, the copolymerization of polybutylene succinate with butylene adipate monomers up to 60% increases chain mobility and biodegradability of the copolymer by lowering both crystallinity and melting temperature [93]. Chen et al. demonstrates that the loading efficiency of daidzein in both the microspheres and in the deposited coatings can be adjusted by varying the processing parameters during microsphere fabrication and electrophoretic deposition process [94]. The authors studied degradation of the deposited multilayers in PBS for up to 14 days. The results revealed that more work is required to further optimize the material structure (both microspheres and coatings) and to comprehensively understand the interaction between drug molecules and polymers used. For example, the degradation rate of the coating should be adjusted in order to avoid severe weight loss during the initial incubation stage meanwhile in the case of bulk material the surface uniformity of free microspheres, should be further optimized to produce smoother microspheres in order to tune the initial burst release effect [94]. Hu J. et al. reported the successful fabrication of nanofibrous scaffolds by emulsion electrospinning of metformin hydrochloride or metoprolol tartrate with PCL or PHBV [95]. Then, the authors evaluated the influence of preparation processes and emulsion compositions (polymer/drug/surfactant) towards the drug release behavior of the scaffolds, together with their morphology, surface, and thermal properties [95]. When compared to blended electrospun nanofibers, in vitro release studies revealed that the emulsion electrospun nanofibers substantially reduced burst release and created a sustained release of drugs. [95].

Another representative example is Chitosan whose degradation process usually begins with random splitting of β-1,4-glycosidic bonds (depolymerization) followed by *N*-acetyl linkage (deacetylation) [49]. Simultaneously with chitosan chain scission, at the same time, the cleavage and/or destruction of its functional groups (amino, carbonyl, amido, and hydroxyl) can happen [50]. It is solubilized in solutions with acidic pH.

In vivo, chitosan is degraded by several enzymes, mainly by lysozyme producing non-toxic oligosaccharides which can be then excreted or incorporated to glycosoaminoglycans and glycoproteins [51]. In vitro degradation of chitosan via oxidation, chemical, or enzymatic hydrolysis reactions are commonly used methods for the preparation of low molecular chitosan under controlled conditions [52]. Youling Yuan et al. evaluated bonding, degradation, and bone cell growth on titanium coated with chitosan of different degree of deacetylation and from different manufacturers [96]. The results suggest that several factors, such as degree of deacetylation, molecular weight, and origination, are important for coating properties and may also be important to in vivo tissue response [96].

These findings were also confirmed by the study presented in Ref. [97] which agrees that chitosan degradation rates can be easily tuned by the degree of deacetylation [97].

Several studies have reported the use of drug-loaded chitosan-based dressings/bandages for wound healing and treatment of open-skin wound infections [98,99], burn infections [100], and/or surgical-site infections [101]. The dressing could deliver the loaded drug into the wound area, thus providing a sustained antimicrobial activity.

#### 3.2.2. Polymers with Limited Biodegradation Capacity

Cellulose and its derivatives (ether and ester) can render distinct drug delivery property patterns, even immediate, delayed, or sustained release [102,103,104,105].

In the study of Yallapu et al., CUR-conjugated Cellulose nanocrystals (CNCs) (5.2 nm) revealed a significant cellular uptake and anticancer activity on prostate cancer cells compared with free CUR [106].

Solanki and Thakore used cellulose cross-linked with PCL and lactic acid (LA)/glycolic acid (GA)/dimethylol-propionic acid to encapsulate felodipine for controlled delivery [107].

Furthermore, the capability of cellulose-based polymers as bio-filler and hydrogel matrix are of great importance for developing sustainable additive manufacturing [103]. Thus, 4D printing of cellulose-based materials could be considered essential in biomedical applications, especially for drug delivery and/or soft robotic applications [105].

#### 3.2.3. Synthetic Polymers

PGA, a synthetic polymer, can degrade by its carbonyl groups cleavage under hydrolytic or enzymatic conditions [55]. Its degradation product is l-lactic acid. Its aliphatic ester bonds are responsible of its hydrolytic instability. PGA is fully biodegraded by the organism within 4 months, but its mechanical properties almost disappear after 6 weeks [55,56,57,58].

Shuqiang Liu et al. demonstrated that the degradation rate and cycle of drug release from suture (Ciprofloxacin) can be tuned by adjusting the proportion of PGA and PCL [65]. One notes that in the degradation process the suture gradually degraded from the coating materials to the inside fibers [65].

Another example shows the degradation mechanism of the polyanihidre, PSA. In the study of Cui Zhixiang et al., poly (sebacic acid) diacetoxy terminated (PSADT) tablets with a circular cross-section were formed using a compression molding device, and then immersed into PBS for in vitro degradation experiments [69]. When the device thickness is greater than the critical sample thickness and the rate of hydrolysis is more rapid than the rate of water diffusion into the device, the degradation mechanism is mainly realized by surface erosion and controlled by varying the amount of hydrophobic or hydrophilic monomers [69,70].

Zhuoling Deng et al. proved that the degradation kinetics of polymeric thin films differ significantly from bulk materials, as interfacial effects become dominant. Therefore, it is crucial to investigate their kinetic separately. In their work, PSA is used as a model system for a quantitative degradation study. Two degradation kinetic regimes are observed when plotting the relative layer thickness determined by ellipsometry and surface plasmon resonance (SPR) against the degradation time, which corresponds to two different rates of erosion. The results revealed that in the case of PSA, the degradation rate could be thickness dependent [73].

Another study of Stefan et al. [71] is dedicated to the degradation behavior (in SBF at 37  °C) of MAPLE PC based on PSADT, in dynamic regime. In general terms, bulk polyanhydrides have a hydrophobic backbone where anhydride bonds are subjected to hydrolysis because they are very water-labile; these polymers degrade with a surface erosion. The depolymerization reaction is too quick to allow water to enter the matrix, which erodes over time. In the exemplified study of P.C. degradation, weighing measurements of degraded samples confirmed slower degradation tendency with increasing initial PSADT amount. 

Birgit Romberg et al. summarized in their paper the current status of PAA-coated liposomes. The results regarding circulation kinetics and enzymatic degradability of the PAA-coating revealed that the PC are degradable by proteases, which is beneficial for reducing the risks of accumulation in vivo [78].

Visan et al. examined the long-term degradation of coatings based on various formulations of PCL-polyethylene glycol blends (PCL-blend-PEG) under simulated conditions. During the first year, PCL ester groups are hydrolytically cleaved. Next, intracellular degradation occurs. Meanwhile, bulk PEG degrades under physiological conditions by hydrolytic and enzymatic cleavage of the phosphate bonds in the backbone to phosphate, alcohol, and diols. It is soluble in aqueous solutions. Results proved that the alteration of blend coatings is strongly governed by the properties of the main bulk polymer constituents being enhanced by the increase of PEG content in the polymeric composite coatings [18].

## 4. Degradation Mechanisms

A reference work (based on the available books [30,69,108,109] and papers in the field) that provide basic information on degradation mechanisms would constitute a useful starting point in future selection of synthesis deposition method, properties, and applications of biodegradable polymers.

The degradation process can happens to polymers [108] either by physical (bulk, degradation, erosion, disintegration, dissolution) or through chemical routes (enzymatic or hydrolytic). Physical degradation of the polymers can be a result of the chemical changes due to the surrounding conditions.

The next section comprises the description of degradation process driven by chemical and physical phenomena. Depending on polymer features and the exposure place in the body, the degradation rate could be tuned in the desired way, also taking into account the biological interactions.

### 4.1. Physical Degradation

**Figure 5:**
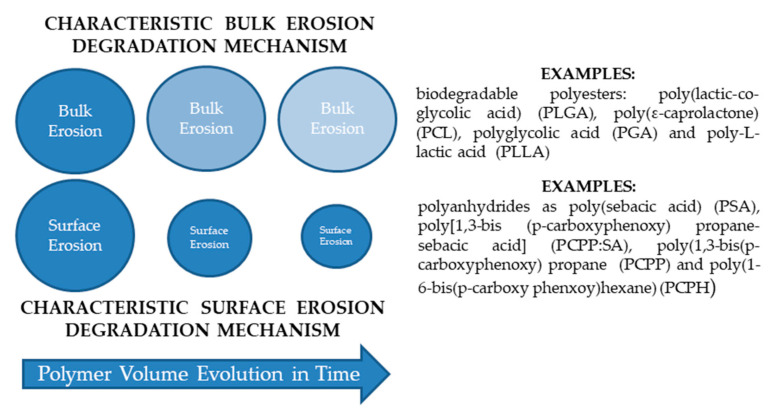
Schematic representation of evolution in time of surface erosion versus bulk erosion.

Basically, physical degradation of polymers in biological environments involves two complementary processes: degradation (refers to polymer chains scission cleaved into oligomers and monomers) and erosion (represents the loss of material due to monomers and oligomers leaving the polymer), respectively (Figure 5) [30].

Bulk degradation implies a faster degradation inside than at the surface of polymer [28]. In the case of an ideal bulk erosion, the rate depends on the total amount of the material which is lost from the entire polymer volume at the same time due to water penetrating the bulk, while remaining a constant size during the degradation process [109]. Some polymers (especially the hydrophilic ones) that exhibit this characteristic bulk erosion degradation mechanism are: the biodegradable polyesters, Poly (lactic-*co*-glycolic acid) (PLGA), PCL, PGA, and Poly-l-lactic acid (PLLA). Depending on the monomers used and their molecular weights, the degradation time of these polymers, widely used in drug delivery applications, varies from a few months to several years [109].

In ideal surface erosion, the rate is proportional to the surface area [34]. Concretely, the erosion of polymer surface becomes smaller while keeping their original geometric shape during the degradation process (biomaterial is lost only from the polymer matrix surface) [34]. Some typical examples of polymers which exhibit characteristic surface erosion degradation mechanism are polyanhydrides as e.g., PSA, poly (1,3-bis (p-carboxyphenoxy) propane (PCPP), poly [1,3-bis (p-carboxyphenoxy) propane-sebacic acid] (PCPP:SA), and poly (1-6-bis (p-carboxy phenxoy) hexane) (PCPH). Such polymers exhibit generally a hydrophobic behavior wherein water cannot penetrate easily into the bulk. The erosion time in this case can vary from few days to several years [69].

The disintegration process is related to particles fragmentation to an acceptable size (depending on the required application) [28].

Another discussion interferes in clarifying the dissolution term which is attributed to a solution of macromolecules, constituting a polymeric biomaterial in a liquid medium [28].

### 4.2. Chemical Degradation

Of all degradation types, the chemical degradation is particularly pertinent for polymers used in biomedical applications (the observed type of degradation being dependent on the type of bonds comprising the polymer, typically within the backbone) [110]. Molecular chain scission can be initiated as mentioned earlier, either passively by hydrolysis or actively by enzyme-catalyzed hydrolysis [111]. Oxidation process may also occur [110]. Chemical degradation causes the main deterioration of polymeric chains by a random cleavage of covalent bounds, depolymerization, or cross-linking of linear polymers, interfering with regularly order chain and with crystallinity, finally resulting in a decrease of mechanical properties [112].

Enzymatic degradation is considered by the catalytic action of enzymes under abiotic conditions, meanwhile hydrolytic degradation is defined as degradation identified as resulting from hydrolytic cleavage of macromolecules [28]. While enzymatically degradable polymers contain hydrolytically labile/unstable/capable to change bonds, these bonds are too stable under physiologic conditions and also require an enzymatic catalyst to undergo degradation [113]. However, numerous bonds (e.g., anhydride, ortho-ester, ester, urea, urethane/carbonate, and amide) will undergo passive hydrolytic degradation under physiologic conditions [110]. There are illustrated the specific cases when degradation process is accompanied by a decrease in molar mass (e.g., vinyl polymers, polyamides) and the situations when degradation means changes in chemical structure (e.g., polymers with aromatic rings in the main chain). The process can also be accompanied by cross-linking [28].

Cross-linking is an important factor because the obtained polymers are generally mechanically strong and resistant to wear, heat or attack by solvents [114]. The degree of cross-linking that occurs is determined by the percentage of polymer chains that are interconnected in the network [115].

Some studies revealed that polymer nanocarriers can reversibly deform under stress while maintaining structural integrity or transmembrane diffusivity [115]. Cross-linking may sometimes present some adverse effects exhibiting a decrease in mechanical properties and exploitation durability [114]. It is reported that the mechanical properties of non-mineralized and mineralized collagen fibers are significantly affected by the cross-linking method [116]. Furthermore, the degradation process is influenced by polymer composition and molecular structure, polydispersity, hydrophilicity or hydrophobicity characteristics, surface area, or crystallinity.

The chemical degradation category also covers the degradation in a biological environment, which could be defined as a gradual breakdown of a biomaterial mediated by a specific biological activity. When polymers are exposed to body fluids they may undergo changes in their physico-chemical properties as a result of chemical, physical, mechanical, and biological interactions between the biomaterial and the surrounding environment [30]. Therefore, the manipulation of the degradation process is fundamental not only to modulate the duration of a biomaterial inside the body but also to control the biocompatibility or the drug release [30].

The degradation process of a polymeric biomaterials inside the body is generated mainly through oxidation, hydrolytic, or enzymatic mechanisms.

In Table 2 the principles of above-mentioned general mechanisms are collected.

All polymers which contain hydrolysable bonds (e.g., glycosides, esters, anhydrides, orthoesters, amides, carbonates, ureas, or urethanes) exhibit a hydrolytic degradation mechanism [130]. As mentioned before, the hydrophilic characteristics of polymers support the modulation of the hydrolytic degradation rate. Biomaterials such as PEG have a high solubilization rate [131].

In the case of aliphatic polyesters such as PLGA, the acid products accelerate degradation due to autocatalysis [132]. Superoxide could accelerate the degradation of aliphatic polyesters by the cleavage of ester bonds via nucleophilic attack of O_2_^−^ [117,133]. It was also reported that polyurethanes are attacked initially by neutrophils which secretes reactive oxygen species and HOCl, one of the most oxidative compounds [134]. In the presence of cholesterol esterase enzyme, polyurethane degradation is about ten times greater than in the absence of the enzyme [135].

The action of particular enzymes prevents enzymatic degradation of natural origin polymers. In the case of hyaluronic acid in mammals [136], the degradation was carried out by theaction of three enzymes: hyaluronidase, β-*D*-glucuronidase, and β-*N*-acetyl-*D*-hexosaminidase. As for chitin derivatives, lysozyme is the enzyme involved in their degradation inside the body [117].

### 4.3. Factors Affecting Degradation Mechanisms

There are a large number of factors that influence polymers degradation capability and their overall rate of degradation. Among them, one should mention the copolymer composition [137], morphology [138], autocatalysis by acidic degradation products inside a matrix [139], presence of drugs [140], and preparation technique [141]. Hydrophilicity–hydrophobicity balance, the structure and molecular weight of the polymer can also predetermine the degradation behavior [142]. Moreover, the environmental conditions such as temperature, the presence of other additives in the polymer, pH, humidity, oxygen, the amount and the microbial strains, salinity, or exposure to external influences (e.g., UV, X-ray, γ-ray, ion beams, or mechanical strain) could also have a great influence [142].

For example, temperature and glass transition temperature (*T*_g_) was evidencing to affect the rate of hydrolysis of a PGA/PLLA copolymer (about 25%–30%), the rate increasing with increasing temperature [143]. An increase in temperature enhances the degradation rate and it is more pronounced when is above the *T*_g_ of the polymer [144].

Depending on the polymer chain type arrangement (e.g., linear, branched or cross-linked [145]) and its crystallinity or amorphous nature, the degradation rate can be altered [145].

Additionally, molecular weight proved to produce effects on polymeric degradation rate. Thus, a high molecular weight (*M*_w_) polymer degrades slowly compared to a low molecular weight polymer. However, the high *M*_w_ PLLA degrades faster due to its amorphous nature [146]. The lower *M*_w_ nanocarriers degraded more quickly, resulting in mass loss, pH decline, and a rapid drug release rate in vitro. In the case of PLGA, the degradation and the drug release are dependent on the polymer *M*_w_ [147].

Additionally, cross-linking causes packing of the polymer, make it impermeable to water which will slow down the degradation process [148].

Morphology, size and geometry also represent important parameters. A large active surface that interacts with surrounding environment can accelerate the degradation rate of the polymer.

During the bulk degradation of as-polymerized PLLA, a rapid decrease of *M*_w_ and tensile properties was observed. This could be explained by the morphology of the material and the presence of thermal stresses and subsequent generation of micro-cracks [149].

Large size plates degrade faster and heterogeneous than thinner films. A linear relationship between the degradation rate and the particle size was found, with larger particles degrading fastest. For smaller particles, the degradation products formed within the particle can diffuse easily to the surface, while for the larger ones the degradation products have a longer path to the surface. Thus, autocatalytic degradation of the remaining polymer material can occur [150].

It was shown [151] that devices with large surface area degrade faster.

Copolymer composition and monomer structure and composition are things to be considered when evaluating B.P. and/or P.C. Thus, it was shown that the increase in glycolide content accelerates polymer degradation [152].

Monomers containing hydrolyzable bonds (e.g., anhydrides) undergo rapid degradation compared to polymers with ester bonds. The order of degradation of different chemical groups is anhydride > ester > amide [153].

Copolymers can be random, alternative, graft, or block copolymers. The possibility of forming a particular type of copolymer depends on the reaction conditions during polymerization and the monomers reactivity ratio. The degradation behavior of alternative and random copolymers consisting of the same molar ratio of co-monomers can be significantly different [154].

There are available numerous combinations of polymeric blends available, which are including a wide range of matrix and dispersed phase bulk biomaterials (e.g., they vary in component compatibility/incompatibility, partial miscibility, or size and shape of inclusions). Thus, another aspect to be considered is deformation of polymer blends which includes a number of micromechanisms (e.g., crazing, shear yielding, different forms of cavitation). These deformability effects may affect decrease in mechanical properties and exploitation durability [155].

Another aspect to be considered is the hydrophilicity/hydrophobicity nature of studied material. The results revealed that the hydrophilic polymers show faster degradation due to the higher water absorption or diffusion into the polymer. Thus, preparation technique could be of great importance. PDLA and PLGA spray-dried particles degrade faster than particles prepared by solvent evaporation [151]. In addition, differences in processing conditions, during fabrication, may also play a role. Polymers that undergo bulk erosion degrade faster than those that undergo surface erosion [156].

On the other hand, pH should not be neglected. Polymers immersed in neutral pH medium undergo slower degradation or solibilization than in acidic and/or alkaline pH [153].

Crystallinity (or *T*_g_) offers interesting information about the degradation process. Crystalline polymers undergo slower degradation compared to amorphous ones [146].

Autocatalysis is another interesting example to be considered to induce an effect on degradation process. There is a study where it is shown that degradation is uneven and takes place faster in the center compared to surface [152].

Additionally, the type of drug may influence the polymer degradation, which may vary from bulk to surface erosion. Similarly, polymeric devices with higher drug loading show higher initial release compared to those with less drug loading [157].

The structural polymeric characteristics such as flexibility, chemical linkages, degree of cross-linking, composition, morphology, microstructure, polarity, and extent of crystallinity have a strong influence on the degradability of polymers. In general, polymer degradation is accelerated by polarity, superior hydrophilicity in the backbone or end groups, inferior crystallinity, lower average molecular weight, and a smaller size of the finished device [145]. The influence of various factors on the degradation process of most widely used polymers is presented in Table 3.

An important factor that affects the degradation process of polymeric films used for drug delivery applications is the interaction between the polymeric matrix and the drug. Besides the drug embedment, the physico-chemical properties are a critical parameter with strong effect on the polymer degradation process and implicitly in the drug release [171]. Hydrophilic drugs accelerate the polymer degradation by facilitating the water penetration in the system and creating highly porous polymer networks upon drug leaching. In contrast, lipophilic drugs slow down the polymer degradation by hindering the water diffusion into the matrix [171]. In the case of acidic drugs, one can notice an accelerated degradation due to the acid catalysis justified by a faster hydrolysis of ester bonds [171]. For basic drugs, the autocatalytic effect of acidic chain ends can be minimized or eliminated based on two effects: (1) base catalysis of ester bond cleavage and (2) neutralization of carboxyl end groups of polymer chains [171]. Thus, the degradation can be either accelerated or slowed down depending on the relative importance of the two observed effects [171,172].

The literature examples emphasize once again an important aspect to be considered in the design of controlled drug delivery systems, namely that the degradation rate of a polymer depends to a large extent on easily controllable factors, as discussed above.

## 5. Deposition Techniques for Polymeric Thin Films Fabrication

### 5.1. Sample Development Methods

Thin film deposition methods can be classified as chemical or physical [33]. Basically, chemical methods (e.g., chemical vapor deposition, sol-gel) involve gas-phase or liquid-phase chemical reactions. Physical methods typically include evaporation, sputtering (ejection of materials from a target followed by condensation to form films), and spraying [173].

In order to obtain better quality polymeric thin films, one should point out some requirements regarding the substrate and processing methods (control of several parameters during and after deposition) which are essential when choosing the deposition technique. Some techniques which require high processing temperatures (e.g., thermal evaporation) proved unsuitable for some biopolymers [32]. Other aspects to be considered are related to the proper deposition rate, cost, and potential for scaling-up the deposition method [32]. It must be borne in mind that the overall behavior on surfaces is usually different to what is expected from bulk behavior. Even in the case of multiple processes (used to deposit the same source material onto the same substrate), the selection of optimal parameters is of key importance in the achievement of the final properties and structure of the coating [32].

In the following, specifics of coating development with the advantages and specifics of different P.C. and MD applications (insisting on drug delivery ones) are concisely addressed. Taking into account that the deposition process is dependent upon several factors (e.g., substrate nature, operating temperature, deposition rate) it is understandable that thin film properties (e.g., thickness, mechanical properties, or surface chemistry) synthesized by different methods differ greatly [33]. Thus, the deposition technique choice requires a systematic study and comparison [173]. Moreover, the combination of the different deposition techniques (chemical and physical) can realistically enable the exploration and expansion of existing techniques for the fabrication of future films and coatings [173].

In the case of dip-coating method, viscosity of the polymer-solvent solution, the deposition speed or substrate nature affects thickness and the adhesion strength between film and substrate [32] which is an important factor in degradation tests [33].

Up until now, the drawbacks of Langmuir-Blodgett coatings limited their use and impeded the industrial application. Their mechanical and thermal stability is quite low [174]. The polymerization improves stability towards mechanical, thermal, and environmental attack by inducing structural reorganizations, but unfortunately, it can lead to defects in the multilayers (e.g., by shrinkage) [174]. These problems can be minimized by appropriate molecular design [174].

Nahir Dib et al. [175] evaluated in their work, simple and Albendazole (ABZ)-dendron mixed films synthesized by Langmuir-Blodgett technique, the composite coating being proposed as surface mediated antitumoral delivery systems without cytotoxic effects [175].

Dib et al. used Methyl thio-5-propyl-1H-benzimidazole2-yl carbamate (Albendazole, ABZ) as a model anthelmintic therapeutic agent, proving its good potential as an antitumor agent. Formation and characterization of pure (dendrons) and composite (drug-dendron) stable and reproducible monolayers, and their transfer to solid substrates, was reported also in their work [175].

The sol-gel technique is a classic example of substrate-dependent technique. In particular, new perspectives are opened by the so-called in situ processes where the inorganic phase is produced in the presence of a polymer or a monomer [176]. Concretely, polymer to gel process involving mild synthesis conditions (e.g., Pechini method (citrate gel) or evaporation of the solution of the water soluble in the initial solution) can allow the coating of substrates in polymeric form at a low temperature and bioencapsulation of relevant functional biomacromolecules [176]. Besides the advantages (e.g., simpler equipment, low cost, homogeneous films preparation, or reduced densification temperature) exhibited by the P.C. synthesized by sol-gel technique, there still remains some important difficulties to be overtaken (e.g., low wear-resistance and a poor coating adhesion which can often lead to ”peel off” phenomenon) [173].

The incorporation of drugs (Chlorpheniramine maleate, theophylline, and famotidine mixed with Opadry amb II^®^ or Kollicoat IR^®^) into various polymeric compositions using spin coating technology to screen amorphous solid dispersion film formation for oral applications was reported by Albarahmieh E. et al. [177].

Combining spin coating and breath figure process, Thiruselvam Ponnusamy et al. developed a single stage process aiming to obtain porous thin films with incorporated drugs. Both surface and bulk features of porosity were further characterized by SEM and the degradation pattern of PC was examined in PBS. The authors also emphasized that the addition of a small amount of PEG into PLGA facilitates ingress of water into the structure, suggesting that the delivery can be modulated [178]. Salicylic acid (highly water soluble) and ibuprofen (sparingly water soluble) have been chosen as two model drug compounds to characterize the release rates, which proved higher in films of the breath figure morphology rather than in non-porous films [178].

Bulk polymers with in situ gelling behavior can be used alone or in blends for the preparation of drug delivery systems in the form of solid formulations (i.e., polymeric matrices, films) [179]. They can be administered through different routes, to achieve either local or systemic vehicles for drug delivery with further prolonged residence time at the site of action/absorption [179,180].

In another study, a drug delivery system based on chitosan nanoparticles acquired by ionotropic gelation, loaded with teicoplanin, and incorporated in tripolyphosphate (TPP) was investigated. No interaction between teicoplanin and chitosan was evidenced but an increase in nanochitosan size caused by the drug was confirmed [181].

Gandhi et al. also demonstrated the potential of sol-gel technique for potential use as a sustained release device for intracanal drug delivery systems (e.g., chlorhexidine- GELRITE^®^ Gellan (polymer)) [182].

The laser-based technologies are widely used for the fabrication of polymeric coatings, exhibiting controlled thickness, good adhesion to the substrate, low material consumption, and stoichiometry conservation of the growing film [173]. Laser-based thin film deposition techniques (e.g., matrix-assisted pulsed laser evaporation (MAPLE)) are competing with conventional methods used for the development of new materials with tailored properties, a core advantage being the ability to combine multiple materials in different configurations (layered or blended) [183]. The deposition of viable and functional thin films require several key elements: laser depositing system characteristics, the choice of targets and receiver substrates, etc. [183]. Cristescu et al. adopted various protocols in order to perform MAPLE experiments, since pullulan, like most polysaccharides, has poor solubility in common organic solvents. Pullulan, either in bulk [184] or coating form [185], is widely used in drug delivery applications (e.g., epirubicin-loaded cholesterol-modified pullulan self-aggregated NPs [186,187]). Varying the thickness and the composition of the biodegradable polymer in a multilayer implementation, the authors demonstrated that MAPLE processing relies on achieving a modulatory release profile of drug particles [188].

In another study, implants consisting of indomethacin coated with polymeric PEG:PLGA films were produced by MAPLE [189].

Laser-induced forward transfer (LIFT) constitutes a viable alternative to more conventional laser direct writing techniques for microprinting of complex polymers [190], with the additional advantages of presenting higher degrees of integration [191], and avoiding contamination and clogging problems thanks to its non-contact nozzle-free nature [173,192,193].

Another example, the printing of polyvinyl alcohol (PVA) polymer thin films via LIFT was accomplished by using water soluble PVA polymer as a support material for 3D-printed structures [194]. The effects of the laser fluence, the thickness of the donor film, and the collector material on the deposition morphology (shape and size) have been studied. The transfer process in PVA printing by LIFT demonstrated the ability of the deposited material to be solubilized in water, the PVA solubility after the laser irradiation being confirmed by the polymer behavior in deionized water.

The good deposition and adhesion of salmon sperm DNA spots onto poly-l-lysine substrates by LIFT with a pulsed Nd:YAG laser was obtained [195].

Ink jet technology can “print” pharmacologic agents onto small needles, producing a low-cost, painless drug delivery system [196]. In recent studies, ink jet printing has been applied to microneedles-arrays of tiny lancet-shaped polymer needles that are already being used to painlessly deliver vaccines [196].

In the last years, a number of studies on bulk silk [197,198] or silk-based coatings [199] were reported due to polymer capacity of maintaining the functional drugs, tunable degradation, and biocompatibility. In this context, water-based silk fibroin (SF) inks exhibit many attractive features, including the ability to make biochemical compounds available in printed formats, printer-friendly rheological properties, controllable degradation profiles, and good mechanical properties [200]. The bulk polymer properties can be tuned by controlling silk polymorphism and by mixing with other biomaterials (e.g., the addition of keratin, collagen), for supplemental functions [200]. Furthermore, a large variety of signaling molecules (e.g., enzymes, growth factors, cytokines) can be embedded in the silk inks [200].

Lysozyme was used as biologic drug model and was formulated as a solution for printing (hydroxypropyl methylcellulose and chitosan) by thermal inkjet printing [201].

Another example is related to layer-by-layer (LbL) adsorption technique which presents adjustable features (e.g., layer structure, component selection, biocompatibility, degradability, and size/dimension) which can support to overcome any outstanding practical difficulties in delivering therapeutics [202]. Four basic mechanisms of LbL films were reported: (i) disruption of layer interactions, (ii) degradation of the LbL film, (iii) multilayer destruction via physical stimuli, and (iv) phase transitions or polymer rearrangements within the LbL film [202]

Being a simple and inexpensive technique, LbL can also satisfy the strict demands from the economic point of view [203].

A newly proposed LbL self-assembly, known as “instructed assembly (IA)”, showed that the ordered structures of individual molecules can be formed under stimuli (e.g., light, chemical, enzymatic reaction, ligand−receptor interaction) [204]. The LbL technique could also be used to design bioreactors using multi-responsive and multi-compartment capsules for controlled enzymatic reactions [205].

Hu Yan et al. assembled LbL films of chitosan/gelatin pairs where mesoporous silica nanoparticles loaded with b-estradiol are embedded for a nanoreservoir-type drug delivery system onto titanium substrates. B-estradiol release proved responsible for regulating the growth of both osteoblasts and osteoclasts, and the fabrication of such nanoreservoir structures displayed potential to maintain bone homeostasis [206].

Dexamthasone was controlled release from microcapsules produced by polyelectrolyte layer-by-layer nanoassembly. Drug particles encapsulated with up to five double layers were formed by alternating the adsorption of positively charged poly(dimeth-yldiallyl ammonium chloride), negatively charged sodium poly(styrenesulfonate) and depending on the pH positively or negatively charged gelatin A or B onto the surface of the negatively charged drug particles [207].

One major advantage of plasma-enhanced chemical vapor deposition (PECVD) over conventional thermal chemical vapor deposition (CVD) is that the lower temperature in PECVD allows the deposition of layers that cannot tolerate a high temperature, which is respected in most cases of polymers. Moreover, the deposition rate in PECVD is typically higher because the arrival rate of the ionized precursors can be easily controlled [32]. Recently, Alexandra Khlyustova et al. review the technological development in vapor-deposited functional polymer coatings, highlighting their biological applications, including drug delivery and/or tissue engineering [208].

Another example could be plasma polymerization which can offer the opportunity to tailor the surface (either bioreactive or non-reactive) and to change the surface chemistry (possible due to the high retention of functional groups from the organic monomers) [209].

Polymeric bulk materials can be also deposited in form of coatings by vacuum deposition technologies (e.g., thermal reactive evaporation, PECVD, physical vapor deposition, e-beam evaporation, and atomic layer deposition (ALD)). Recently introduced, ALD, compared with conventional CVD methods, allows for a better uniformity and conformity on complex substrates due to the longer lifetime of the precursor molecules to transport and diffuse to the cavities in complicated three-dimensional substrates [210]. It is true that ALD usually do not work on a polymeric material surface [211] but recently Hong Chen Guo et al. developed new nanostructured materials using a binary sequence of self-limiting reactions on curve surfaces beyond planar deposition of thin film. Thus, ALD deposition process can be coupled with precursor permeation into polymer bulk, as well as precursor reaction with polymer chains. These results revealed that polymeric material systems have a large number of various sub-systems based on different polymer chain network, functional groups attached, and interstitial space formed [211].

Vogel et al. demonstrated that ALD can be utilized to slow the mat degradation in both humid and aqueous conditions, from several minutes to multiple weeks, just by controlling the thickness of the deposited Al_2_O_3_ coating on electrospun poly (vinyl alcohol) nanofibers through increased ALD cycles [212]. Thus, changing the rate at which nanofibers dissolve modulates the release of embedded small molecules (ketoprofen) within the polymer matrix from minutes to weeks while reducing the “burst” effect [212].

The feasibility of transforming a drug-containing liquid polymer into a solid hybrid material was demonstrated by Boehler et al. using ALD [213]. While the PEG serves as a dispensing medium for any kind of potential biologically relevant molecules, the subsequent atomic layer deposition of ZnO converts the liquid drug-solution into a solid hybrid layer, forming the storage phase [213]. This solid film can be coated with a thin conducting polymer film serving as a gate-keeper, enabling an active release system for a broad variety of substances [213].

Film deposition by sputtering is predictable and stable and the evaporation process requires a relatively good vacuum to ensure collision-less trajectories of evaporated atoms before condensation. To minimize residual gases that can contaminate the film, a high (10^−5^ Pa) or ultrahigh (<10^−7^ Pa) vacuum may be needed to produce films with a desired purity [209]. The deposition of polymeric coatings by RF magnetron sputtering proved suitable for so-called plasma polymer deposition and can be used for controlled release of antibiotic substances [214]. These type of coatings exhibit a considerably higher levels of cross-linking and branching, as well as an absence of regularly repeating monomer units [215]. Despite their random and inherently complex structure, plasma polymer coatings offer the possibility to fine tune wettability and a bio-adhesive/bio-repellent behavior of deposited surfaces [215].

Another interesting example, the electrostatic powder deposition (ESPD) method, was applied in the development of films for drug delivery. Prasad et al. proposed a technique which allows for a high degree of flexibility in preparation of films with discrete shapes and sizes, without the need for cutting. Films were prepared using PEO, a physical mixture of PEO and acetaminophen (APAP), and co-processed PEO and APAP particles [216]. Additionally, the healing times in the case of ESPD films proved to be significantly reduced compared to films obtained by solvent casting processes (the solvent evaporation can take hours). In other areas, the ESPD is already employed at industrial scale, thus many processing parameters and formulation features [216] were already investigated and part of this knowledge can be translated or expanded and used for pharmaceutical manufacturing [216]. Khalil S. et al. recently reported a research regarding the development of a multi-nozzle deposition system for biopolymers. In order to fabricate three-dimensional structures, three types of nozzle systems have been used for the deposition of sodium alginate from aqueous solutions (with various viscosities) and one for PCL [217]. The system is capable to simultaneously design scaffolds, depositing a controlled amount of a bioactive compound with precise spatial position [217].

We summarized in Table 4 the most widely used techniques for the controlled synthesis of polymer-based coatings used as drug delivery systems.

### 5.2. Degradation Simulation Methods

In vitro degradation behavior can be evaluated by mass loss measurements of weight changes of the sample (e.g., degradable polymers used as coating materials for surface functionalization of metallic implants) at different time intervals. One method could be to place the tested sample in a bioreactor, e.g., a laboratory-controlled system, so that parameters such as SBF/PBS flow rate, pH, temperature, and humidity can be controlled.

To simulate the processes occurring inside human tissues [250], the polymeric-coated samples can be tested under physiological-mimicking dynamic conditions (in different solutions which simulate the body fluids, SBF or PBS, at a temperature of 37 °C) using a manufactured set-up consisting of a multichannel degradation cell [71,251].

Different type of bioreactors (e.g., spinner flask, rotating wall bioreactors, or perfusion systems) could be applied for in vitro PC testing. While spinner flasks [252] and rotating wall [253] bioreactors have been shown to boost in vitro culture conditions by increasing homogeneity of nutrients in the media, perfusion systems [254] exposed cells to shear stress and efficiently enhance nutrient transfer [255]. A bioreactor has the potential to minimize the contamination from bacteria, reduce labor intensity, or the costs through automatization. Moreover, a cell source could be attached to a bioreactor, seeded, and cultured continuously in the closed system. Nutrient and oxygen concentrations could be monitored by the system and the media replacement could be automated [255].

One of the mentioned reactors, designed for testing the polymeric coatings, was reported by Socol G. [71] and represents a reliable method for studying the degradation behavior of polymeric materials, either in bulk or coating form, owing to its simplicity and similarity to the organism conditions. However, the accuracy of the method is not fully controllable [71] due to the statistical process of synthesizing reproducible thin films and also to the small amount of active substance (e.g., drugs, natural antimicrobial agents, etc.) that can be incorporated into the polymeric matrix.

In vivo characterization of polymers used as coating materials for surface functionalization of implants and drug delivery applications is even more challenging due to contributions from adsorbed inflammatory exudates, proteins, and reactive oxygen species which are coming in contact with the polymeric surface [171]. In vivo characterization could be extended to different passive and active drug delivery P.C. systems used in wound, cancer treatment or for antimicrobial applications [256,257,258].

Various animals like mice, rats, rabbits, hamsters, fish (e.g., zebra fish, trout), birds (e.g. chicken), guinea pigs, amphibians (xenopus frogs), dogs, and cats could be used in research for in vivo testing [259].

Various integrated approaches (e.g., computer models, bioinformatics tools, enzymatic screens, modern analytical techniques, data acquisition, and statistical procedures) could be used as alternatives to animal involvement in scientific procedures. Thus, it is required to investigate the theoretical kinetics modeling and perform simulations to predict the properties [171].

For example, [151,167] it was shown by computational modeling that PLLA degradation erosion is sensitive to crystallinity.

## 6. In Vitro Degradation Characterization Methods

According to the ISO 846-97 standard, polymers degradation can be determined either by the visual observations or by the measurement of changes which occurs in mass and physical properties. Depending on requirements, different categories of testing methods are applied (e.g., in vivo simulation and in vitro laboratory tests).

The most employed techniques applied to evidence the degradation of polymeric coatings or stabilization in time are listed below [260].

1. Compositional assessment (e.g., oxidation; hydrolysis; chemical reactions)—the chemical changes resulted due to scission or cross-linking of polymer chains as well as changes in intermolecular forces. These changes can be analyzed by spectroscopic methods, wet-chemical analysis, gravimetric tests, Energy-Dispersive X-ray Spectroscopy (EDXS), or X-ray Photoelectron Spectroscopy (XPS). Fourier Transform Infrared (FT-IR) and RAMAN Spectroscopy are complementary methods commonly used to indicate chain degradation, oxidation, increase of hydroxyl group impact, water adsorption, etc.

The changes observed on FTIR spectra (the disappearance of peaks) as a consequence of degradation were evidenced in the in vitro study performed on PLLA meshes. Thus, FTIR applied to monitor the degradation provided information by chain scissions on both polymer composition and crystallinity. Then, mappings of in vivo degraded PLLA meshes were realized to better visualize their degradation mechanisms [261].

Other spectroscopic techniques could be also used, e.g., with TOF-SIMS. Thus, a good linearity was obtained in the kinetics study of PLLA degradation [170,171]. X-ray Diffraction (XRD) and XPS can yield important information about the structure and chemical composition of polymers and their mechanisms of degradation.

For example, it was found that the hydrolysis of ester bonds proceeds linearly with time. This finding shows that the chemical reaction, rather than water diffusion, is the governing mechanism. The results also show that degradation rate increases with increasing polydispersity [165,166].

Distinct spectroscopic evidence of morphological changes (i.e., an increase of crystallinity) was noticed [168,169,262]. The hydrolytic degradation of polyethylene(terephthalate) (PET) in water occurs preferentially at terminal ester sites, whilst in alkaline solution it is a much more random process [154,157].

Additionally, nuclear magnetic resonance could represent a powerful spectrometric technique which provides information about stability and degradation of the polymers (e.g. degree of acetylation, of amination, or sulfonation [27]. In a study related to the functionalizing the nanocarriers surfaces with a tissue-recognition ligand [27], Nuclear Magnetic Resonance (NMR) analysis confirmed the incorporation of the ligands (PLA- PEG- folic acid and PLA- PEG -biotin conjugates) on the nanoparticles.

2. Morphological changes investigations—study of surface cracking responsible for the change in mechanical properties. Applied techniques: Scanning Electron Microscopy (SEM); Atomic Force Microscop (AFM); Optical microscopy (OM).

Qualitative evaluation of polymer degradation can be performed by SEM, OM (when cracks peel off or holes can be observed) and by AFM which can provide insight into the rugosity of P.C. In the study of Cui Zhixiang et al., AFM and SEM techniques were used to demonstrate that the surface roughness increases with the degradation time. Additionally, it is found that both the number and size of pores increase with the degradation time [69].

In another example, the degradation behavior of polymeric coatings was evidenced by SEM images of the PCL-blend-PEG during immersion in SBF up to 16 weeks correlated with SEM images yielded from electrochemical experiments [18]. The appearance of holes corresponding to eroded areas were observed [18]. It was found that the PEG solubilizes fast, immediately after the immersion, while the PCL degrades slowly over the whole period of time [18,153].

3. Macromolecular properties evaluation-chain length that produces the shrinkage forces leading to surface cracking. Applied techniques: XRD, Differential Scanning Calorimetry (DSC), and Size Exclusion Chromatography (SEC). The study of thermodynamic parameters, such as: thermal (DSC differential scanning calorimetry) or thermomechanical properties analysis (TMA), including *T*_g_, melting point (crystalline, semicrystalline polymers), decomposition point; decomposition can be determined from thermogravimetric analysis (TGA). Dynamic mechanical analysis (DMA) is worth mentioning also and may provide important information that can be used in the development of new products and improvement of those already in the market [164]. For example, *T*_g_ and *M*_w_ decrease as degradation proceeds [152,164].

In the next example, the DSC curves of PSADT samples processed at different temperatures are very similar to that of the unprocessed ones, indicating that the processing method does not induce any additional crystallinity compared to the raw material. Additionally, in the same study it was found that processing temperature has no significant effect on the PSADT degradation rate [69].

Another important standard technique to characterize polymers degradation is determination of the molecular weight reduction by means of gel permeation chromatography or by intrinsic viscosity [263]. Many theories link the drug diffusion coefficient inside degradable polymers to the polymer molecular weight, as small chain molecules offer less restriction for drug diffusion than long chains, thus making the presented above technique of great importance [27].

Thermal properties of the polymeric materials are reported in the work of Flores-Ramırez et al. TGA results on the functionalized hybrid chitosan material with two different stoichiometric molar ratios revealed that the degradation temperatures decrease as the degree of functionalization increases [264].

4. Mechanical properties tests (e.g., tribological measurements; nanoindentation tests) —degradation often corresponds to a transition from a ductile to brittle mode of failure which can be studied by Young’s modulus. One should note that the whole stress -strain behavior is an important indicator of degradation as the Young’s modulus may also increase sometimes due to crystallization, whereas total sigma-epsilon behavior indicates reduced strain, etc.
5. Degradation behavior can be also evaluated by mass loss weights and/or electrochemical measurements. The fact that the metallic implants corrode in the human body remains a challenge, the electrochemical experiments can be also used to induce accelerated degradation effects. Therefore, the control of blending biomaterials can be applied with the pursuit to regulate the corrosion rate and prevent rapid corrosion. However, one should mention that this is an accelerated test method, and the test parameters are quite different from actual environmental conditions.

The degradation study of PSADT was confirmed by mass measurements that the rates of mass loss is almost linear during the degradation process, indicating that a near zero-order degradation kinetics theory holds good for this polymer [69].

Meanwhile, bulk characterization offers information on the macroscopic properties of the biomaterial such as mechanical, solubilization, optical, thermal, or dielectric properties, the surface characterization can reveal critical morphological information for interfacing the implant or drug delivery device with the host tissue.

Additionally, in the lower pH of media conditions, the PLGA was faster degraded generally. The presence of various additives, moreover, affected decrease of pH and slight acceleration of LA and GA detection [156].

A literature survey on polymeric biomaterial characterization techniques used to evidence the degradation processes is given in Table 5.

One should note here that the same techniques for characterizing and highlighting the degradation phenomenon are applied both for bulk material and coatings.

Due to the fact that in the case of coatings where small quantities of material are involved, more attention is needed in order to obtain any form of meaningful data on P.C., the measuring results are often at the limit of detection of the devices. Thus, the low detection limit of the devices in case of coatings exposes them to higher errors, thus making difficult the whole process of characterization of the degradation phenomenon. Although the techniques resolution (e.g., tribological equipment, spectrometric analyzers) has been improved significantly in recent years, a more careful interpretation of these results is still needed.

Moreover, the complete mathematical analysis of the different degradation mechanisms is not fully elucidated [286], here also being place for more detailed studies.

Additionally, the difficulties that appear only in the specific case of thin film degradation strengthen the above-mentioned comments. We refer here to exfoliation of the film (e.g., peel off) which may occur due to poor adhesion to substrate or there may be situations when water can enter under the deposited film so the degradation assessment process can be seriously compromised.

All these observations justify the small number of publications on the degradation of polymeric systems in the form of coatings because these studies involve a higher volume of work compared to bulk materials and leads to the need for greater statistics.

## 7. Outlines and Perspectives

Manipulation of the degradation process is fundamental not only for the tuning of a polymeric biomaterial inside the body but also to modulate the biocompatibility or drug release. This work provides a basic overview of polymer degradation mechanisms, pointing the factors influencing the degradation, the advanced characterization techniques employed to evaluate the degradation, and the most widely used polymers in drug delivery applications. Because degradation processes can significantly differ from system to system, all discussions in this review are always made in comparison: bulk polymeric systems versus thin film form. Understanding the degradation mechanism of polymers (e.g., degradation kinetics, identification of degradation products, influencing factors) is, therefore, of great importance when selecting and designing polymeric systems for desired applications.

The information centralized in this paper will help readers to find out more about this challenging subject and will be a useful reference in the future.

However, it must be borne in mind that polymeric coatings become more important for long term performance of new products develop and a more comprehensive approach must be performed. Furthermore, social and industrial demands for cost effectiveness, lower environmental impact, and high-performance increase the technologies choice impact. For example, in the context of the COVID-19 pandemic, the production of biodegradable gloves coated with drugs that do not encourage the proliferation of the virus may consist as a feasible solution for rubber solid waste disposal problem along with a solution for slowing down the hazard pandemic. Another possible application could be 3D printing of drug delivery implants or even personalized 3D printed implants. In order to improve the production process and succeed to the market penetration, the laboratory-scale or pilot-scale applications restrict should be overcome. Many aspects still need to be considered (the statistical process of synthesizing reproducible thin coatings or the proper amount of active substance that can be embedded into the polymeric matrix) to thoroughly investigate the polymer degradation-based coatings behavior.

## Figures and Tables

**Table 1:** Widely used degradable polymers for drug delivery applications in form of bulk (B.P.) and P.C.

D.P.	D.T.	Model Drug	Reference B.P.	Reference P.C.
**NATURAL Polypeptides** *e.g.,* *Poly (3-hydroxybutyric acid-co-3-hydroxyvaleric acid (PHVB)*	years	Levofloxacin (LEV)	[34,35,36,37]	[38,39,40,41,42]
		Gemcitabine (Gem)		
		Curcumin (CUR)		
**NATURAL** **Proteins** *e.g.,* *Collagen*	Few days-weeks completely degrade	Doxorubicin (DOX)	[43]	[44,45,46,47,48]
**NATURAL Polysaccharides** *e.g.,* *Chitosan*	up to 8 weeks	Indomethacin (IDC)	[49,50]	[49,51,52,53]
		Paracetamol (PaC)		
**SYNTHETIC** **Polyester** *e.g.,* *Poly (glycolic acid) (PGA);* *Polylactic acid (PLA) and their copolymers*	4–8 months	DOX	[54,55,56]	[57,58,59,60,61,62]
		5-fluorouracil (5-FU)		
**Synthetic** **Polyester** *e.g., Poly (ε-caprolactone) (PCL)*	>24 months up to 2–3 years	DOX	[63,64]	[18,65,66,67,68]
		Vancomycin (VaC)		
**Synthetic Polyanhydrides** *e.g., Poly (sebacic acid) (PSA)*	few days to several years	Deguelin (Deg)	[69,70]	[71,72,73,74,75]
		Cisplatin (Cis)		
		MUC4β-nanovaccine (MUC4-vac)		
**Synthetic Polyamides** *e.g., Poly (amino acid) (PAA)*	2–60 days	Glycolic acid (GA)	[76,77]	[78,79,80,81]
**Synthetic** **Polyphosphoester** *e.g., Polyethylene glycol (PEG); Polyethylene oxide (PEO)*	3–250 days	Nobiletin (Nob)	[82,83,84,85]	[18,86,87,88]
		Artemisinin (AMS)		
		Docetaxel(DocT)		
		DOX		
		Indocyanine green (ICG)		

Abbreviations of the table legends: Degradable polymer = D.P.; Degradation time (months) = D.T.

**Table 2:** Exemplification of the general degradation mechanisms type-bulk polymers (B.P.) versus P.C.

D.M.	Basic Steps of the Degradation Mechanism	Reference (B.P.)	Reference (P.C.)
HYDROLYTIC	The first degradation reaction is the hydrolytic scission of the polymer chains which leads to a decrease in the molecular weight [117]. By further hydrolysis, the molecular weight of degradation products is reduced allowing them to diffuse from the bulk material to the surface and then to the solution, causing thus a significant weight loss. Acids, bases, or salts may catalyze the hydrolysis reactions. The biomaterial absorbs water and swells, and degradation will progress from the outside of material towards its interior.	[118,119,120]	[18,121]
OXIDATION	Degradation takes place by oxidation when P.C. are exposed to body fluids and tissues. The oxidative effect of the highly reactive oxygen species (e.g., hydrogen peroxide, superoxide (O_2_), hypochlorous acid (HOCl) and nitric oxide) produced by inflammatory cells (especially macrophages and leukocytes) during inflammatory response to foreign biomaterials may cause polymer chain scission and contribute to their degradation [122].	[123,124]	[125,126,127]
ENZYMATIC	Degradation occurs when enzymes cannot penetrate the inner of the polymer, due to high cross-link density or limited access to cleavage points, forcing the surface or exterior bonds to cleave first [117,128]. Basic interaction steps between enzymes and polymeric chains are: Diffusion of the enzyme from the bulk solution to the solid surface. Adsorption of the enzyme on the substrate, resulting in the formation of the enzyme-substrate complex. Catalysis of the hydrolysis reaction. Diffusion of the soluble degradation products from the solid substrate to the solution [117,128].	[124]	[129]

Abbreviations of the table legends: Degradation mechanism type = D.M.

**Table 3:** Influence of various factors on the polymer degradation.

Factor	Polymer	Reference
TEMPERATURE and *T*_g_	PGA/PLLA, PLGA copolymer	[158,159]
Molecular weight	PLGA, PCL, PEG	[160,161]
Morphology, size and geometry	PGA, PLLA	[162,163]
	Poly (d-lactic acid) (PDLA), PLGA	[159,164]
Copolymer composition	PLGA	[137,165,166]
Monomer structure and composition	Anhydride	[153]
Copolymer type of polymer	PEG	[157]
Hydrophilicity/hydrophobicity	proteins, polyethylene glycol ethers, polyamide, polyacrylic amides	[167]
Preparation technique	PDLA, PLGA	[141,166,168]
pH	PLA, Chitosan, Polyhydroxybutyrate (PHB)	[169]
Crystallinity (or *T*_g_)	PLGA	
Autocatalysis	PDLA	[139,170]
Drug type and drug loading	PLGA	[157]

**Table 4:** Widely used deposition methods for polymeric thin films employed on the degradation subject.

Deposition Method		PC Examples	Reference
**MAPLE**	*Advantages:* Coatings of nanoparticles, application to both organic and inorganic coatings, multilayers and multistructures	pullulan	[218,219,220,221,222,223,224]
		PEG	
	*Drawbacks:* Small covering areas	PCL	
		PEG: PLGA	
**LIFT**	*Advantages:* Patterns with high spatial resolution	polyepichlorhydrine (PECH)	[173,190,191,192,193,218,219,220,225,226,227]
		polyisobutylene (PIB)	
	*Drawbacks:* Limited to patterns; difficulties for large area thin coatings	polyethylenimine (PEI)	
		poly-l-lysine	
**Ink-jet printing**	*Advantages:* Thicker films	PCL/chitosan	[228,229,230,231,232]
		Silk	
	*Drawbacks:* Possible nozzle blockage for composites	hydroxypropyl methylcellulose	
		chitosan	
**Spin coating**	*Advantages:* Simple, uniform coatings	PLGA/ PCL composite	[7,233,234,235]
	*Drawbacks:* Solvent issue during multilayers, adherence		
**Sol-gel**	*Advantages:* Macroporous bioactive scaffold	Chitosan/BG composite	[193,225,228,232,236,237,238,239,240]
	*Drawbacks:* Poor coating adhesion	Polyurethane carboxylated poly (vinyl chloride)	
		gellan	
**Langmuir-Blodgett**	*Advantages:* Monolayers	Glucose	[18,241,242]
	*Drawbacks:* Limited to very thin films	PCL	
**LbL**	*Advantages:* Viscoelasticity/bioactivity	PLGA and alginate	[20,130,136,243,244]
		Chitosan	
		Polysaccharide	
	*Drawbacks:* Involvement of liquid media limit multi-layer assembling (affect interfaces)	Chitosan	
		gelatin	
**Electrostatic deposition**	*Advantages:* Solvent-free coating process	PLGA	[130,193,236,237,245,246,247]
	*Drawbacks*: Limited to single coating	polysaccharide	
**Multi-nozzle Deposition Manufacturing**	*Advantages:* Porous structures	PLA/tricalcium phosphate	[217,248,249]
	*Drawbacks:* Possible nozzle blockage for composites	PLGA	

Abbreviations of the table legends: Relevant examples of polymeric coatings used for drug delivery applications = PC examples.

**Table 5:** Characterization techniques used to study polymer degradation behavior.

Polymer	Degradation Parameters	Characterization Technique	Drug	Reference
PDLA	*M*_w_, *T*_g_, Thermal changes	DSC, SEM	Prednisolone	[265,266,267]
Polyesters	Absorbance of peak quantify changes in the concentration of degradation products and thus to provide indications regarding the kinetic constant of the hydrolysis reaction.	FT−IR	Antihypertensive drugs	[268,269,270]
PCL-blend-PEG	Weight measurements	Bioreactor, Electrochemical tests; SEM; FT-IR	Paclitaxel	[18,66,271]
PET	Changes in concentration	IR	5-fluorouracil and 6-thioguanine	[272,273,274]
PLLA	Polymer structure and composition	Computational modeling	lidocaine	[275,276,277]
SF-PSADT	Weight measurements	FT-IR, XRD, SEM, bioreactor	cisplatin	[71,278]
LA and GA in PLGA	pH	HPLC	MEK1/2 inhibitor GDC-0623	[279,280]
Copolymer of lactide/caprolactone	Chemical composition, molecular weight, morphology	Raman Spectroscopy	vancomycin	[281,282,283]
PLLA	Surface molecular weight and end-group	TOF-SIMS	Ciprofloxacin	[65,284,285]

## Data Availability

The data presented in this study are available on request from the corresponding author.

## Abbreviations

5-fluorouracil	(5-FU)
Acetaminophen	(APAP)
Arginine glatiramer acetate	(GA)
Artemisinin	(AMS)
Atomic Force Microscopy	(AFM)
Atomic layer deposition	(ALD)
Bulk	(B.P.)
Cellulose nanocrystals	(CNCs)
Chemical vapor deposition	(CVD)
Cisplatin	(Cis)
Curcumin	(CUR)
Degradable polymer	(D.P.)
Degradation mechanism type	(D.M.)
Degradation mechanism	(D.M.)
Degradation time (months)	(D.T.)
Deguelin	(Deg)
Differential Scanning Calorimetry	(DSC)
Docetaxel	(DocT)
Doxorubicin	(DOX)
Dynamic Mechanical Analysis (DMA)	Electrostatic Powder Deposition (ESPD)
Energy-Dispersive X-ray Spectroscopy	(EDXS)
Fibroblast growth factor 2	(FGF2)
Fibronectin	(FN)
Fourier Transform Infrared Spectroscopy	(FT−IR)
Gel Permeation Chromatography	(GPC)
Gemcitabine	(Gem)
Glycolic acid	(GA)
Grazing Incidence X-ray Diffraction	(GIXRD)
High-performance liquid chromatography	(HPLC)
Human Growth Hormone	(hGH)
Hypochlorous acid	(HOCl)
Indocyanine green	(ICG)
Indomethacin	(IDC)
Infrared Spectroscopy	(IR)
Instructed assembly	(IA)
Lactic acid	(LA)
Laser-induced forward transfer	(LIFT)
Layer-by-layer	(LbL)
Levofloxacin	(LEV)
Mass spectrometry	(MS)
Matrix assisted pulsed laser evaporation	(MAPLE)
Medical devices	(MD)
Metformin hydrochloride	(MH)
Metoprolol tartrate	(MPT)
MUC4β-nanovaccine	(MUC4-vac)
Multi Angle Light Scattering	(MALS)
Nobiletin	(Nob)
Nuclear magnetic resonance	(NMR)
Paracetamol	(PaC)
Phosphate buffered saline	(PBS)
Plasma-enhanced chemical vapor deposition	(PECVD)
Poly (sebacic acid) diacetoxy terminated	(PSADT)
Poly [1,3-bis (p-carboxyphenoxy) propane-sebacic acid]	(PCPP:SA)
Poly (1,3-bis (p-carboxyphenoxy) propane	(PCPP)
Poly (1-6-bis (p-carboxy phenxoy) hexane)	(PCPH)
Poly (3-hydroxybutyric acid-*co*-3-hydroxyvaleric acid)	(PHBV)
Poly (amino acid)	(PAA)
Poly (D-lactic acid)	(PDLA)
Poly (lactic-*co*-glycolic acid)	(PLGA)
Poly (sebacic acid)	(PSA)
Poly (ε-caprolactone)	(PCL)
Polyepichlorhydrine	(PECH)
Polyethylene (terephthalate)	(PET)
Polyethylene glycol	(PEG)
Polyethylene oxide	(PEO)
Polyethylenimine	(PEI)
Polyglycolide or Poly (glycolic acid)	(PGA)
Polyhydroxybutyrate	(PHB)
Polyhydroxyvalerate	(PHV)
Polyisobutylene	(PIB)
Polylactic acid	(PLA)
Poly-L-lactic acid	(PLLA)
Polymeric coatings	(P.C.)
Polyvinyl alcohol	(PVA)
Scanning Electron Microscopy	(SEM)
Scanning Transmission Electron Microscopy	(STEM)
Silk fibroin	(SF)
Simulated body fluid	(SBF)
Size Exclusion Chromatography	(SEC)
Surface plasmon resonance	(SPR)
Thermogravimetric Analysis	(TGA)
Thermomechanical Properties Analysis	(TMA)
Time-of-Flight Secondary Ion Mass Spectrometry	(TOF–SIMS)
Transmission Electron Microscopy	(TEM)
Tripolyphosphate	(TPP)
Ultraviolet–visible spectroscopy	(UV-VIS)
Vancomycin	(VaC)
X-ray Diffraction	(XRD)
X-ray Photoelectron Spectroscopy	(XPS)
β-tricalcium phosphate	(β-TCP)
